# Pattern of infectious diseases in northern Iran: An approach to internal medicine management

**DOI:** 10.22088/cjim.12.3.275

**Published:** 2021-04

**Authors:** Mohammad Ali Jahani, Zohreh Alinasab, Maysam Rezapour, Melodi Omrani Nava, Ghahraman Mahmoudi

**Affiliations:** 1 Social Determinants of Health Research Center, Health Research Institute, Babol University of Medical Sciences, Babol, Iran; 2 Hospital Administration Research Center, Sari Branch, Islamic Azad University, Sari, Iran; 3 School of Nursing and Midwifery, Amol, Mazandaran University of Medical Sciences, Sari, Iran

**Keywords:** Contagious, Infectious diseases, Disease process, Infectious

## Abstract

**Background:**

Despite the development of their prevention and treatment, infectious diseases cause high mortality, many disabilities and inadequate living conditions worldwide. The aim of this study was to evaluate the pattern of infectious diseases in northern Iran with an approach to internal medicine management.

**Methods:**

This cross-sectional research was conducted in 2019 on all 7095 infectious diseases patient records that referred to Ghaemshahr Razi Hospital, Mazandaran Province, Iran during 2012-2018. A checklist prepared by investigator was used to collect the data. The extracted data were coded and entered into SPSS 22 and analyzed using K^2^ and independent t-test at p<.05.

**Results:**

The mean age of the study patients was 29.7±26.4. 4372 (61%) of the cases were males and the mean duration of hospitalization was 41.6±33.5. Age was significantly correlated to infectious diseases (P=.001). Gastroenteritis was the most common infectious disease among the men and women with 2442 (60.5%) and 1594 (39.47%), respectively. Based on the Pearson's correlation test, the relationship between leptospirosis, brucellosis, pulmonary tuberculosis, shigellosis, sepsis and infectious mononucleosis with gender, habitation, admission mode, discharged mode and age was significant (p<.05).

**Conclusion:**

As the high frequent diseases were gastroenteritis, leptospirosis, brucellosis and sepsis and an increasing trend was in the prevalence of gastroenteritis, leptospirosis and lung tuberculosis, health system managers should consider training courses, preventive strategies, real-time interventions, increased hospital bed rate for patients with infectious diseases and so on.

In spite of the major development in the treatment and prevention of infectious diseases, they are yet the main reasons for mortality, inability and low-level life condition among millions of people in the world (1). Infectious diseases can directly cause one in four deaths around the world (2). They generally occur at the start of life and can be prevented (2). It has been proven that microbial resistance has increased in parallel with the development in antimicrobial agents and some diseases such as tuberculosis, cholera and acute rheumatic fever have resuscitated despite the fact that it is conceived that they had been eradicated completely in the modern world (3). The statistics on mortality from infectious diseases does not determine the real statistics on mortality from infectious agents as much mortality from infectious agents are not categorized as the ones caused by infectious diseases, including among other diseases caused by infectious agents in cancer and secondary cardio-vascular diseases (4).

It has been proven that infectious agents play a role in arteriosclerosis. The data show the role of certain infective agents in immune diseases such as rheumatoid arthritis and inflammatory bowel diseases (5). Infectious diseases are of the most common causes of death and disability in Iran (6). Development of urbanization and change in the industrial lifestyle as well as change in age pyramid and continuous aging of people in the future result in the increase of the prevalence of infectious diseases in the country (7). Annually, nine million people in the world suffer from tuberculosis and 1.5 million die from it. Of these, 360000 suffer from tuberculosis and AIDS (8). During warm seasons, viral infections occur more frequently and the use of contaminated food supplies causes infectious diseases (9).

Ataey and Jafarvand reported a downward trend and slowness in tuberculosis mortality in Iran (10). Hajari et al. reported an upward trend in brucellosis in Iran over a 10-year period (2004-2013) (11). Based on a study by Metanat et al., the epidemiology of infectious diseases in 45 recent years has decreased in southeastern Iran, with Golestan and Sistan and Balouchestan provinces with the highest rate in this regard (12). Sharma et al. have stated that tuberculosis has threatened the population over the past two decades that new and immediate preventions strategies are needed to prevent it (13).

Nowadays, epidemiological studies of infectious diseases are needed for controlling many infectious diseases, monitoring changes in infectious disease patterns, some infectious disease resuscitation and infectious origin of some acute diseases and not completely eradication of infectious diseases (14). These studies can highlight trends in these diseases and criticize the social and human damages associated with their prevalence (15). These studies can also help determine the ways of distributing and preventing them (6). Therefore, considering the importance of this subject, the purpose of the present study was to evaluate the pattern of infectious diseases in northern Iran with approach to internal medicine management.

## Methods

This cross-sectional study was conducted in 2019 on all electronic records of 7095 infectious disease patients who were referred to Ghaemshahr Razi Hospital, Mazandaran Province, Iran in 2012-2018. Razi Hospital is located in Ghaemshahr City of Mazandaran province and is a teaching hospital. There are 253 beds in the hospital, of which 44 are infectious and 17 are emergency support beds. It is the reference center for infected patients in the province of Mazandaran.

No age, gender or race limit were taken into account. Inclusion criteria included records of hospitalized patients diagnosed by ICD10. Exclusion criteria were incomplete or diagnostic records based on ICD10. ICD-10 is the 10th revision of the International Statistical Classification of Diseases and Related Health Problems (ICD), a medical classification list provided by the World Health Organization (WHO) and contains codes for diseases, their signs and symptoms, abnormal findings, complaints, social circumstances, and external causes. Infectious diseases are categorized under A. 

Tuberculosis can be diagnosed by a chronic cough accompanied by bloody sputum, fever, night sweats and weight loss. Diagnosis of active tuberculosis can be done by radiology (x-ray) and microscopic examination of microbiological culture of body fluids(23). Symptoms of leptospirosis include fever, severe headache, muscle aches, especially in the legs, wrists, and back, and conjunctival hyperemia. The definitive diagnosis of leptospirosis is carried out by serologic antibody tests. In sepsis, fever, discomfort, weakness or confusion are the symptoms of the disease. There is also tachycardia and tachypnea. If left untreated, it can make breathing difficult which can cause some mental problems (15). Infectious mononucleosis is associated with fever, sore throat, neck lymphadenopathy and fatigue. Gastroenteritis or gastrointestinal inflammation is usually diagnosed based on the symptoms such as blood in the stool or in people who have been exposed to food poisoning, with diarrhea, vomiting, abdominal pain and muscle cramps(38). Brucellosis is diagnosed with symptoms such as sudden shivering, general body aches, loss of appetite, fatigue, fever, back pain, joint pain, and Wright and Combs Wright laboratory tests, 2ME, and CRP (31).

A checklist prepared by the researchers was used for data collection including items on gender, age, habitation, admission and discharge modes, hospitalization days, etc. After confirming the topic in the Research Ethics Council of Islamic Azad University, Sari Unit, Mazandaran, Iran and taking the ethical code of IR.IAU.CHALUS.REC.023.1397, researchers began to collect needed data by coordination with hospital managers in the Health Information Department. The extracted data were coded and entered into SPSS 22 and analyzed by chi-square test and independent t-test.

## Results

As shown in table 1, among the patients studied, 4372 (61%) were males with the mean age of 26.4±29.7 years old and the mean length of hospitalization was 41.6±33.5 days. 6764 (95%) and 6880 (96%) of the cases were admitted at the clinic and discharged by physician, respectively. The relative frequency of gastroenteritis, leptospirosis, brucellosis, and sepsis was 43.39, 13.12, 5.86 and 3.89 in the studied patients, respectively (table 2). As shown in table 3, the mean age of infected patients was 26.4±29.7 years old. The least mean of hospitalization duration was 3.1±3.6 days in shigellosis and the highest mean of hospitalization duration was 11.3±10.2 days for pulmonary tuberculosis. The first to third ranks belonged to gastroenteritis with 4038 cases (56%), leptospirosis with 977 cases (13.7%) and pulmonary tuberculosis with 451 cases (6.3%), respectively. The mean age rates of pulmonary tuberculosis and leptospirosis cases were significantly higher than those of other diseases (P=.001). Taking into account the Pearson's correlation test, the relationship between leptospirosis, brucellosis, pulmonary tuberculosis, shigellosis, sepsis and infectious mononucleosis with gender, admission mode, discharged mode and age was significant, except leptospirosis vs. inpatient duration (table 3). 

**Table 1:** Demographic information on inpatients with infectious diseases

**Year**	**Age (M**±**SD)**	**Gender (M±SD)**		**Hospitalization days (M**±**SD)**	**Having insurance**	**Admission mode (via clinic)**	**Discharge mode (via physician's order**
		**Male**	**Female**				
2012	29.3±27.6	577(57%)	435(43%)	17.6±36.5	986(97%)	911(90%)	973(96%)
2013	29.4±26.6	587(59%)	407(41%)	13.4±12.5	969(97%)	831(83%)	973(98%)
2014	28.8±27.1	566(61%)	361(39%)	43.5±38.5	901(97%)	897(96%)	896(96%)
2015	30.5±27.8	727(64%)	408(36%)	43.12±27.4	179(95%)	1119(98%)	1083(95%)
2016	31.5±26.7	628(62%)	384(38%)	38.5±33.5	933(99%)	981(98%)	976(97%)
2017	29.9±25.2	756(63%)	417(37%)	24.6±42.4	1174(98%)	1191(99%)	1154(96%)
2018	26.4±25.3	531(63%)	311(37%)	43.4±45.4	834(99%)	834(99%)	825(97%)
Total	26.4±29.7	4372(61%)	2723(39%)	41.6±33.5	6936(97%)	6764(95%)	6880(96%)

**Table 2:** The relative frequencies of infectious diseases among patients referred to Mazandaran Infectious Referral Center

**ICD10 Code **	** Title**	**Count**	**%**
A09	Other gastroenteritis and colitis of infectious and unspecified origin	3079	43.39
A27.9	Leptospirosis, unspecified	931	13.12
A23.9	Brucellosis, unspecified	416	5.86
A41.9	Sepsis, unspecified	276	3.89
A16.2	Tuberculosis of lung, without mention of bacteriological or histological confirmation	248	3.49
A16.9	Respiratory tuberculosis unspecified, without mention of bacteriological or histological confirmation	202	2.85
A03.9	Shigellosis, unspecified	157	2.21
B02.9	Zoster without complication	142	2.00
A49.9	Bacterial infection, unspecified	129	1.82
B27.9	Infectious mononucleosis, unspecified	125	1.76
B01.9	Varicella without complication	104	1.47
B18.2	Chronic viral hepatitis C	84	1.18
B16.9	Acute hepatitis B without delta-agent and without hepatic coma	65	0.92
B15.9	Hepatitis A without hepatic coma	43	0.61
A37.9	Whooping cough, unspecified	38	0.54
B17.1	Acute hepatitis C	31	0.44
B00.9	Herpesviral infection, unspecified	31	0.44
B00.2	Herpesviral gingivostomatitis and pharyngotonsillitis	29	0.41
A27.0	Leptospirosis icterohaemorrhagica	21	0.30
B17	Other acute viral hepatitis	18	0.25
A38	Scarlet fever	14	0.20
B08.0	Other orthopoxvirus infections	13	0.18
A18.2	Tuberculous peripheral lymphadenopathy	11	0.16
B67.9	Echinococcosis, other and unspecified	10	0.14
B55.9	Leishmaniasis, unspecified	9	0.13

**Table 3:** The relationship between variables and types of infectious diseases

**Variable**		**Lung tuberculosis**	**Leptospirosis**	**Gastroenteritis**	**Brucellosis**	**Shigellosis**	**Sepsis**	**Infectious mononucleosis**
Gender	Male	346	747	2444	244	77	158	77
	Female	105	230	1594	121	82	119	52
	Total	451	977	4038	365	159	277	129
p-value		<0.001	<0.001	o.o3	0.035	0.001	0.011	0.651
Admission Mode	Offices	19	9	15	0	0	44	0
	Clinics	417	948	3894	357	152	217	123
	others	15	20	129	8	7	16	6
	total	451	977	4038	365	159	277	129
p-value		<0.001	<0.001	<0.001	0.03	0.236	<0.001	0.275
Discharge mode	Physician's order	435	965	38	6880	156	259	128
	Patient's permission	16	12	141	216	3	18	1
	Total	451	977	179	7096	159	277	129
p-value		0.025	<0.001	0.012	0.271	0.390	0.001	0.130
Age		45.3±19.7	45.7±18.0	24.7±26.5	42.3±19.0	28.4±24.7	46.9±33.2	21.3±8.9
p-value		0.001	0.001	0.001	0.001	0.627	0.001	0.001
Hospitalization duration		11.3±10.2	5.2±3.2	3.9±7.1	7.4±4.4	3.6±3.1	6.9±6.7	4.6±1.9
p-value		0.001	0.491	0.001	0.001	0.001	0.001	0.08

**Figure 1:**
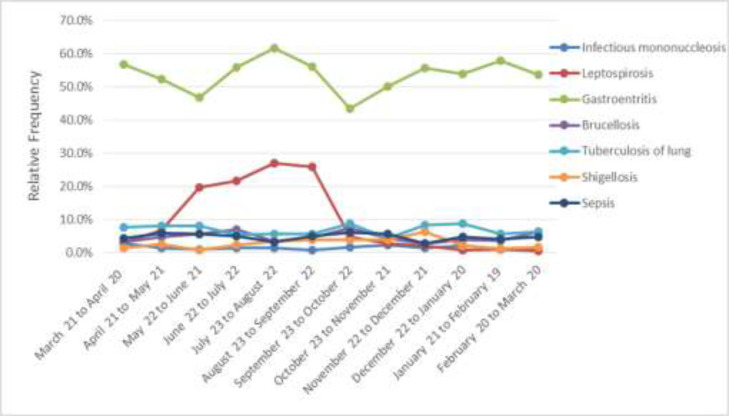
The relative frequencies of the most common hospitalization of the infectious disease patients by year between 2012 and 2018

**Figure 2:**
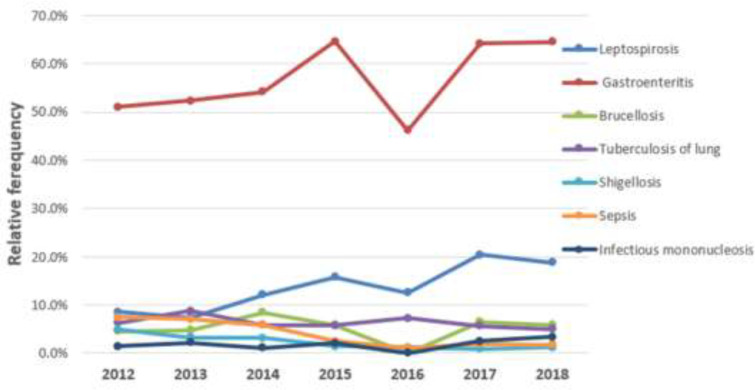
The relative frequencies of the most common hospitalized patients with infectious diseases per year from 2012 to 2018 (n= 7095)

Figure 1 shows the relative frequency of the most common infectious diseases among the hospitalized patients studied in a month. As can be seen, gastroenteritis patients were hospitalized for significant period of the time compared to other patients. Figure 2 shows the relative frequencies of the most common hospitalization of the infectious disease patients by the year between 2012 and 2018. As can be seen, patients with gastroenteritis have been hospitalized in considerable frequencies compared to other patients, too. In addition, the frequency of leptospirosis patients increased from 2012 to 2018.

## Discussion

The findings showed that the infections were different among the males and female patients, with the exception of infectious mononucleosis and sepsis. The age of patients was significantly associated with related infectious diseases, with exception of shigellosis. Patients' length of stay in hospital has a significant relationship with their related infectious diseases, with the exception of leptospirosis and mononucleosis. Trends in leptospirosis, gastroenteritis and long-term tuberculosis are increasing and trends in sepsis, shigellosis, infectious mononucleosis, and brucellosis are decreasing. The highest frequency of hospitalized illness was recorded in the summer of 2018. The highest incidence of leptospirosis occurred between March and August.

In this study, the mean age of patients with an infectious disease was 26.4±29.7. In Eini P’s , Keramat’s study, the high rate of infection was observed in men between ages of 40-8 (16). Gastroenteritis, leptospirosis and pulmonary brucellosis were the major infectious diseases resulting in hospitalization Eini P, Keramat reported pneumonia, pulmonary tuberculosis and gastroenteritis as major infectious diseases resulting in hospitalization(16). These differences indicate that having information on common seasonal infectious diseases in each region can be useful in making health decisions. The incidence of leptospirosis was on the rise in northern Iran and higher among men than women. The positive cases were higher in hot months (during agricultural season). This disease has had an increasing trend in recent years and by a changed pattern, it has become a common and epidemic disease in urban societies with inappropriate and weak hygiene level (17) during hot seasons(18). Katelaris et al. identified it as a common occupational disease among raspberry workers due to ongoing contamination of to their scratched hands with mice infected leptospirosis (19).

In Colombia (20), leptospirosis was mostly found in men older than 18 years. Rafiei et al.found that this was work-related disease that is higher among men than women, generally limited in northern Iran (21). In our study, leptospirosis was higher in men than women. However, Noorimanesh et al. in Astara, Guilan province found no significant difference between men and women in this respect (22). The reasons maybe that in Mazandaran most agricultural activities are done by men, but in Guilan, they were generally done by men and women altogether.

The trend in the incident of pulmonary tuberculosis was on the rise and infected men were higher than infected women. This pattern has been detected in the Russian Federation (23) and Brazil (24). However, there was down trend in both European Union countries (25). The difference may be the infection can be prevented by comprehensive interventional programs and screening plans as well as people's increased awareness of the disease and accessing cheap related drugs in European countries. Lifestyles are at work in this difference, too. The tuberculosis is projected to continue to trend into 2020 as a major source of future diseases. If the existing control programs are not improved, one billion people will be infected with a new tuberculosis infection with 150 million affected patients as well as 36 million deaths (26). Rahmanian et al.(27) and Valizadeh et al.(28) found higher rates of infection in men possibly due to some socio-economical, biological characteristics as well as different rates of men and women's presence in social affairs and activities. 

There has been declining trend in the incidence of brucellosis in northern Iran. The highest rate of brucellosis occurred in eastern Mediterranean (29). Nguna et al. in Uganda found that the incidence of brucellosis was related to small families consuming domestically produced milk (30). Tumwine et al.found that the possibility of brucellosis incidence was elevated among rural men consuming raw milk products (31).

The mean age of patients suffering from brucellosis was 42.3 in our study. However, Tumwine found it as >60. Based on our results, brucellosis has shown the same trend through years and its incidence has decreased further since 2014. Mostafavi and Asmand observed a downward trend in brucellosis and stated that high awareness of people and the use of healthy milk products can be of key factors in this regard(32). We found a downward trend in the impact of shigellosis in northern Iran. The high incidence of the disease can be seen in developing countries(33) and regions with low hygiene indicators. In a study carried out in 6 countries in Asia, the incidence of the disease was high among children and individuals with >40 years old. The mean age of patients with shigellosis was 28.4 years old in our study probably due to some differences among the populations studied.

In our study, sepsis tended to decline. It was the third leading cause of death from infectious diseases after infectious lung diseases and AIDS (34). Sepsis is the most common cause of mortality in the ICUs because of its high incidence rate and related problems, including septic shock (35). Infectious mononucleosis was significantly related to gender and its trend was declining in northern Iran. In Western societies, it is spread to adult through kissing and in developing countries by touching saliva-contaminated toys and tools (36). In one study in southeast Iran, its incidence increased with increasing age (37). The first incidence is high in the first decade of life and is most common in the United States among 15-24 years age groups (38).

Our findings showed an increasing trend in gastroenteritis in the years studied, with a higher incidence rate among men and during warm months. 39. Hatami et al. found that its incidence was higher among women and during cold months with 60% of patients under 27 years of age(39). Because they studied norovirus-induced gastroenteritis that is common in cold months, their results may be limited. While records obtained from hospitals are considered as appropriate sources of epidemiological information, there are limitations to this study. First, hospital admissions are selective depending on the severity of diseases and admission policies different from some of other hospitals in Mazandaran province, because it is a referral center for infectious diseases. Second, because hospital data are not designed for the study, so they may be incomplete, illegible and missing. Third, the diagnostic quality of diseases varies through a physician and clinical service. Fourth, considering the relative frequencies of these diseases as the prevalence is wrong, because the catchment area population of these diseases are not defined and varied from other cities in Mazandaran province and even neighboring provinces. 

Based on our results, the high frequent diseases were gastroenteritis, leptospirosis, brucellosis and sepsis and an increasing trend was seen in the prevalence of gastroenteritis, leptospirosis and lung tuberculosis and a decreasing trend in sepsis, shigellosis, mononucleosis and brucellosis. As Mazandaran province is along the passengers/travelers’ way and considering an increase in trips during summer and the communicability of infectious diseases, it is needed that travelers are trained in preventing these diseases. Several brochures are available for different age groups in this province. Health authorities may use the results of the study and tend to provide more hygiene facilities for rural areas. Careful laboratory tests must be conducted at the time of admission and complete certainty recovery at the discharge time. The further research is necessary to study the high frequencies of infectious diseases and to design appropriate programs to achieve a better controlled situation.

 Studying more variables was not possible in this study because of the lack of some information on the studied patients, such as their occupation and educational level and their potential effect on the epidemiology of the studied infectious diseases.
